# Genome-wide analysis, expansion and expression of the NAC family under drought and heat stresses in bread wheat (*T*. *aestivum* L.)

**DOI:** 10.1371/journal.pone.0213390

**Published:** 2019-03-06

**Authors:** Claire Guérin, Jane Roche, Vincent Allard, Catherine Ravel, Said Mouzeyar, Mohamed Fouad Bouzidi

**Affiliations:** UMR 1095 Génétique, Diversité et Ecophysiologie des Céréales, Université Clermont Auvergne, INRA, Clermont–Ferrand, France; Huazhong University of Science and Technology, CHINA

## Abstract

The NAC family is one of the largest plant-specific transcription factor families, and some of its members are known to play major roles in plant development and response to biotic and abiotic stresses. Here, we inventoried 488 NAC members in bread wheat (*Triticum aestivum*). Using the recent release of the wheat genome (IWGS RefSeq v1.0), we studied duplication events focusing on genomic regions from 4B-4D-5A chromosomes as an example of the family expansion and neofunctionalization of *TaNAC* members. Differentially expressed *TaNAC* genes in organs and in response to abiotic stresses were identified using publicly available RNAseq data. Expression profiling of 23 selected candidate *TaNAC* genes was studied in leaf and grain from two bread wheat genotypes at two developmental stages in field drought conditions and revealed insights into their specific and/or overlapping expression patterns. This study showed that, of the 23 *TaNAC* genes, seven have a leaf-specific expression and five have a grain-specific expression. In addition, the grain-specific genes profiles in response to drought depend on the genotype. These genes may be considered as potential candidates for further functional validation and could present an interest for crop improvement programs in response to climate change. Globally, the present study provides new insights into evolution, divergence and functional analysis of *NAC* gene family in bread wheat.

## Introduction

As sessile organisms, plants have developed diverse strategies to modulate their development and growth according to environmental signals such as day/night alternation or daily temperature variations [1]. At the molecular and cellular levels, the early mechanisms of response to these stimuli include signal perception by receptors, followed by signalling cascades involving changes in membrane permeability, protein phosphorylations by protein kinases or regulations of the target protein expression by transcription factors (TFs) [2]. About 6–8% of the plant genome is allocated to the coding of more than 1,500 TFs [3] of which 45% belong to plant-specific families [4]. Among more than 80 TF families, AP2/EREBP, bZIP, MYB/MYC, NAC and WRKY families are known to be strongly involved in the response to biotic or abiotic stresses in plants [5–16]. The NAC (**N**AM (No Apical Meristem)–**A**TAF (Arabidopsis Transcription Activation Factor)–**C**UC (Cup-shaped Cotyledons)) family is one of the largest groups of plant specific TFs, and has been described in several plant species. In the model plants, Arabidopsis and rice, this family includes 117 and 151 members, respectively [17]. NAC family members have also been reported in *Brachypodium distachyon* (118 members) [18], soybean (101 genes) [19], maize (157 members) [20], durum wheat (168 members) [21], and more recently in Fragaria × ananassa fruits (112 members) [22].

A typical NAC protein contains a highly conserved DNA-binding domain, measuring ~150 amino acids, and located in the N-terminal region. This domain is subdivided into five subdomains named A to E [23]. Subdomains A, C, and D are highly conserved. Subdomains C and D are probably involved in the binding of the NAC protein to its DNA target [24]. An additional poorly conserved transcription regulatory region is present in the C-terminal region of the protein and acts as a transcriptional activator [25] or repressor [26] of target genes. The C-terminal regulatory region often contains simple amino acid repeats and regions rich in serine and threonine, proline and glutamine, or acidic residues [27]. While the majority of the NAC TFs were found as soluble proteins in the nucleus, some NAC have been identified as membrane-bound TFs. They are classified as membrane-related and named NTL (NTM1-like or ‘NAC with transmembrane motif 1’-Like) TFs. For example, nine NTL were identified in durum wheat [21] and at least 85 and 45 in Arabidopsis and rice respectively [28].

Expression of NAC family genes is modulated during plant development and in response to biotic and abiotic stresses [27]. NACs are one of the largest families of genes with very diverse functions. The first *NAC* gene was described as related to the shoot apical meristem development and the position determination of meristems and primordia [29]. The NAC family regulates many developmental and growth processes including flowering [30], root development [25], leaf senescence [31], secondary cell wall biosynthesis [32], cell division [33], and seed development [34]. The NAC family is also related to grain yield and quality. For example, a reduced expression of *OsNAP* in rice and *TaNAC-S* in wheat increases the grain yield by delaying leaf senescence [35–38]. Moreover, NAC proteins have also been shown to be involved in response to biotic stresses such as pathogen infection [39], hypersensitive response-like cell death [40], and abiotic stresses including salt, drought, and temperature [41–43]. Due to their key role in abiotic stress signalling in plants, NAC TFs could be used to improve crop plant tolerance to unfavourable environmental conditions. This is particularly important for bread wheat (*Triticum aestivum)* because the yield of this major crop, which constitutes the diet basis of millions of people, is constrained by abiotic stresses.

Bread wheat is a hexaploid species. Its genome is composed of three subgenomes AA (originated from *T*. *urartu*), BB (from *Aegilops speltoides*) and DD (from *Ae*. *tauschii*) [44]. In a recent publication, Borrill *et al*. [45] carried out an inventory of the TF coding genes in wheat using published TGAC gene models [46], with an emphasis on the NAC family. They identified 574 (453 high and 121 low-confidence) *NAC* genes belonging to eight subfamilies. More recently, a high-quality version of the wheat genome (cv Chinese Spring) assembly was released by the International Wheat Genome Sequencing Consortium (IWGSC) [47]. In this study, we used this last version to analyse the genomic organization and the expansion of the NAC family. We identified 488 (460 high and 28 low-confidence) *NAC* genes, named *TaNAC*, in the three subgenomes A, B, and D of bread wheat. We established the correspondence (one-to-one) between the two annotations (488 *vs* 574) yielding a consensus subset containing 361 identical sequences. The expression profiles of these 361 identical *TaNAC* genes were monitored in different tissues (grains, spikes, leaves and roots) or in response to different abiotic stresses (heat, drought, and heat + drought combination), based on the data available on the www.wheat-expression.com platform [48]. In addition, 23 representative *TaNAC* genes were selected to investigate their response to drought stress using two contrasting wheat cultivars at two developmental stages (220 and 450 degree days (°Cd) after anthesis). Our results provide valuable information about *TaNAC* candidate genes to improve drought stress tolerance in wheat.

## Materials & methods

### Meta-analysis of the NAC family in bread wheat

#### Survey of *TaNAC* in the wheat sequence

For TaNAC retrieval, the annotated proteins in the database of the International Wheat Genome Sequencing Consortium (IWGS RefSeq v1.0) [47] were searched using the HMMER2 program implemented in Unipro UGENE 1.25 [49] and by querying the NAM motif (PF02365, generated by alignment of 587 seed sequences) downloaded from Pfam.

The full-length sequences obtained with an E-value cut-off e^-1^ were scanned for NAC domain superfamily (IPR036093) using InterProScan (http://www.ebi.ac.uk/interpro/search/sequence-search, S1 Table) [50]. The combination of HMMER2 and InterProScan searches led to a final set of 488 TaNAC proteins, whose 460 are high-confidence and 28 are low-confidence. The *TaNAC* genes were mapped on the wheat pseudomolecule from the IWGS Consortium to determine their chromosomal coordinates and visualized using MapGene2Chromosome V2 (http://mg2c.iask.in/mg2c_v2.0/). Homoeologous genes were determined using their chromosomal coordinates.

To create a high quality consensus set of *TaNAC* genes, a reciprocal homology search was performed between the TGACv1 [46] and the IWGS RefSeq v1.0 [47] cDNA databases (including high and low-confidence sequences), using Usearch program [51]. This query led to the identification of common sequences between the *TaNAC* gene set of Borrill *et al*. [45] extracted from TGACv1 cDNA database and the set of 488 *TaNAC* sequences extracted from IWGS RefSeq V1.0. To minimize the fortuitous detection of homology, the final set of protein-coding sequences in both annotations was used as a query. When reciprocal homology search identified the same pair of *TaNAC* genes in both directions, they were considered as homologs.

#### The NAC family structuration in bread wheat

To study the structuration of the TaNAC family and confirm the homoeologous groups, a phylogenetic study was performed. The maximum likelihood method available in MEGA7 software [52] was used to construct an unrooted phylogenetic tree based on the alignment of the amino acid sequences of the common set of sequences established. Alignment was performed using the MUSCLE alignment algorithm [53]. The tree obtained was the consensus of 500 single trees provided by bootstraps, gaps/missing data treatment: partial deletion, model/method LG model, rates among sites: gamma distributed with invariant sites (G).

To explore the contribution of gene duplication to the expansion of the NAC family in bread wheat, we focused on flanking sequences of each *TaNAC* gene. For each locus containing a *TaNAC* gene, 25 Kb on either side of the gene were collected. When several *TaNAC* genes are separated by less than 25 Kb, the window for sequence retrieval was extended to the 25 Kb beyond the leftmost and rightmost *TaNAC* genes. Homologies between nucleotide sequences were obtained using YASS, a Blast-like tool [54] with an e-value threshold of e^-30^. The homology search results were then parsed and sequences with at least 80% identity over more than 2 Kb were considered duplicates. Figures showing these duplications were generated using CIRCA (OMGenomics, http://omgenomics.com/circa/).

To monitor the correlation between expression profiles of duplicated genes, Pearson correlation coefficients between *TaNAC* genes were calculated using the R software (http://www.R-project.org, R development Core Team, 2008).

#### Expression of *TaNAC* genes

The expression profiles of *TaNAC* genes were monitored in different tissues (grains, spikes, leaves, and roots) [55] or in response to different abiotic stresses in leaves (heat shock, polyethylene glycol-induced drought, and a combination of both at a young stage) [56], based on the RNA seq data available on the www.wheat-expression.com platform [48]. Briefly, raw expression data were downloaded and analysed using the R package limma [57]. Raw expression data corresponding to the entire wheat genome were first filtered to remove weakly expressed genes (genes are retained if they are expressed at a counts-per-million (CPM) > 0.5 in at least two samples). The expression of retained genes were then normalized using TMM and Voom methods. Finally, expressions were compared between samples and those genes that displayed an absolute log Fold Change (logFC) >1 with an adjusted p-value < 0.05 were deemed Differentially Expressed and noted DEG. Finally, *TaNAC* genes were retrieved from this set of DEG genes. Hierarchical Clustering of *TaNAC* DEG was conducted using Mev software [58] with euclidean distance and average linkage.

On the basis of this meta-analysis, 23 *TaNAC* genes which displayed large fold changes in their expression were selected to assess their response in wheat plants cultivated in field conditions and submitted to drought.

### Expression analysis of 23 *TaNAC* candidate genes in field-grown stressed wheat plants

To gain insights into the function of wheat *NAC* genes in response to abiotic stresses, we decided to assess the expression patterns of 23 selected *TaNAC* genes in plants subjected to drought stress in field.

#### Plant material and field drought treatment

Field drought treatment was performed on the Pheno3C plant phenotyping platform. This four automatic rain shelters facility is able to generate contrasted water regimes by protecting four “stressed” plots from rainfall, while four “control” plots are managed under natural weather and irrigated if necessary to prevent water stress. The eight plots are distributed over a homogeneous three hectares area and minimum distance between two plots is 30 m. Each plot (22 m x 25 m) is subdivided into 96 micro-plots (1.9 m x 1.2 m) where different genotypes were sown. Among those genotypes and based on local expert knowledge and previous trials, two modern bread wheat genotypes (Allez-y (2011, Limagrain) and Altigo (2007, Limagrain) were selected as stress sensitive and stress tolerant cultivars respectively on the basis of yield impact under dry conditions. Soil volumetric water content (SWC) was recorded hourly with CS 655 (Cambell Scientific, Logan, UT, USA) sensors installed at four depth (10, 35, 50 and 75 cm) at three locations per plot. Total soil water content was calculated by integrating sensor values up to 100 cm depth (average soil depth of the plots). Average soil field capacity and available water capacity are respectively 570 and 280 mm. From those values and daily measurements of SWC, a water stress factor (Ks) was calculated from Allen *et al*. (1998) [59]. A Ks value below 1 is considered as an indicator of water stress that increases in terms of severity when the Ks value decreases.

All plots were sown on 11/04/2016, and managed following local agronomic practices. The stress was applied starting on 03/24/2017 by sheltering the stressed plots. At anthesis (05/20/2017), control plots had received 130 mm more rain than stressed plots, leading to a Ks of 0.85 and 0.36 in the control and stressed plots respectively, defining a very moderate stress in the control and a severe stress in the stress plots. Converted degree-days (°Cd) were calculated as the sum of mean daily temperature (base 0) from anthesis. For the present experiment, three micro-plots per genotype and per hydric conditions (biological triplicates) located in two adjacent blocks were used at both sampling dates. The samplings occurred at 250°Cd after anthesis (end of grain cell division) and 450°Cd after anthesis (middle of the linear phase of grain filling). At 250°Cd, water stresses were very moderate (Ks = 0.91) and very severe (Ks = 0.27) in the control and stressed plots respectively. At 450°Cd, water stresses were moderate (Ks = 0.6) and very severe (Ks = 0.2) in the control and stressed plots respectively. For each sample, three spikes and three leaves were taken randomly and frozen immediately in liquid nitrogen prior to subsequent RT-qPCR analysis. Yield components of each plot were also assessed. Spike number per m^2^, which is determined before anthesis [60], is a good indicator of the plant stress response before flowering, reflecting the stress impact on the plant tillering. This variable was determined manually by counting all spikes on two 1.9 m rows per micro-plot. Grain yield was measured at maturity with an experimental plot combine harvester.

#### RNA isolation and relative quantification of candidate genes

To quantify the expression of the 23 candidate genes, a total RNA extraction was performed on the collected samples, using the protocol described by Capron *et al*. [61] for grain samples, and the TRIzol protocol according to the manufacturer’s instructions (Invitrogen) for leaf samples. Reverse-transcription (RT) was performed on 2.5 μg of total RNA, using the Thermo Scientific Maxima First Strand cDNA Synthesis Kit according to the manufacturer’s instructions. A RNAse H (Thermo Scientific 18021–071) treatment was performed after the reverse transcription.

When selecting the 23 *TaNAC* genes, only one member per homoeologous group was retained. Then, their specific expression profile was studied using Real Time-quantitative Polymerase Chain Reaction (RT-qPCR) and by designing primers specific to the coding sequence of the considered homoeolog (listed in S2 Table), with PrimerQuest (https://eu.idtdna.com/PrimerQuest). Amplification efficiencies of primer pairs designed were tested to keep only those with an efficiency comprised between 90 and 110% (S2 Table).

Real Time-quantitative Polymerase Chain Reactions (RT-qPCR) were carried out in a 15 μl volume containing 25 ng of cDNA, 7.5 nM gene-specific primers and 7.5 μl of LightCycler 480 SYBR Green I Master (Roche Diagnostics #04887352001), on a LightCycler 480 system (Roche) on the Gentyane platform, INRA de Crouël, France (http://gentyane.clermont.inra.fr/). The thermal cycle used was done as follows: a pre-incubation at 95°C for 10 min, 40 amplifications cycles of 95°C for 10s, optimal temperature of the primer for 15s, and 72°C for 15s.

Relative target gene expression was determined by the 2^-ΔΔCt^ method [62] using Ta2776 (RNase L inhibitor-like protein) for normalization [61]. A principal component analysis was carried out using the expression data of the whole set of 23 genes across all samples and the R package ggfortify [63]. For each gene, a Kruskal-Wallis test was performed to uncover differences between samples.

## Results

### Inventory and description of the NAC TF family in bread wheat using the IWGS RefSeq v1.0 database

The HMM profile of the Pfam NAC domain (PF02365) was used as a query to identify *TaNAC* genes in the most recent version of the annotated wheat genome released by the IWGSC RefSeq v1.0, which contains the latest assembly of the wheat cv *Chinese Spring* pseudomolecule.

The initial HMM search identified 501 putative TaNAC sequences that were manually curated for the presence of the NAC domain (IPR036093) signature using the Interproscan platform. Thirteen sequences were discarded, giving a whole set of 488 full-length sequences (460 high-confidence and 28 low-confidence sequences) containing the NAC domain (S1 Table).

This set was compared with the sequences identified by Borrill *et al*. [45] using the TGAC database. This analysis provided a robust subset of 361 common sequences, showing a single hit between the two databases (S3 Table). The combination of the two annotations improved the knowledge on the *TaNAC* genes present in this subset, particularly in terms of chromosomal location. For example, no information on the chromosomal location of the TraesCSU01G137200 gene was available from the IWGS RefSeq v1.0 database, but it was found to be identical to the TRIAE_CS42_2AL_TGACv1_093618_AA0283710 gene in the TGAC database mapped on the chromosome 2A. Similarly, among the 13 non-mapped sequences of the TGAC database, 12 were identical to already mapped IWGS sequences. Finally, 359 *TaNAC* genes among the 361 consensus sequences have been mapped on the 21 bread wheat chromosomes, the two remaining sequences did not have chromosomal location information. Chromosomes 2 and 7 contain the highest numbers of *TaNAC* genes with 89 and 81 members respectively, which represent 24.8% and 22.6% of the total. On the contrary, chromosome 1 carries the lowest number of *TaNAC* sequences, with only 17 genes (4.7%) (S1 Fig). The *TaNAC* genes are evenly distributed among the subgenomes A, B, and D with a similar proportions (34.5%, 33.5%, and 32.0% respectively) indicating that the expansion of the NAC family predates the bread wheat appearance through interspecific hybridization.

*TaNAC* genes are mostly distributed next to the telomeric regions *vs* centromeric one (S1 Fig). Furthermore, most of these genes are tightly linked and lay within clusters. For example, TraesCS4B01G328600, TraesCS4B01G328700, TraesCS4B01G328800, TraesCS4B01G328900, and TraesCS4B01G329100 are close on chromosome 4B within 347 Kb. Similarly, the *TaNAC* genes denoted TraesCS4A01G419900, TraesCS4A01G420000, TraesCS4A01G420100, TraesCS4A01G420200, TraesCS4A01G420900, TraesCS4A01G421000, TraesCS4A01G421100, TraesCS4A01G421200, and TraesCS4A01G421300 are tightly linked and lay within 796 Kb.

The genomic length of the sequences within the whole set of 488 *TaNAC* genes, ranged from 273 to 15074 base pairs, corresponding to protein sequences from 91 (TraesCS2B01G556700LC.1) to 1030 amino acids (TraesCS5B01G550800.1) with a mean length of 370 amino acids. One-fifth of the sequences (*i*.*e*. 100 sequences out of 488) are intronless. On the other hand, one sequence (TraesCS5B01G550800.1) contains 16 exons. The majority (214 sequences, 44%) contain three exons (S2 Fig). It is interesting to note that eight sequences, representing three homoeologous groups, contain two NAC domains (TraesCS2A01G363700, TraesCS2B01G381700, TraesCS2D01G361500; TraesCS3A01G269900, TraesCS3B01G303800, TraesCS3D01G269600; and TraesCS2A01G328100, TraesCS2D01G334800) (S1 Table).

### Evolution by duplication of the TaNAC family

**Fig 1:**
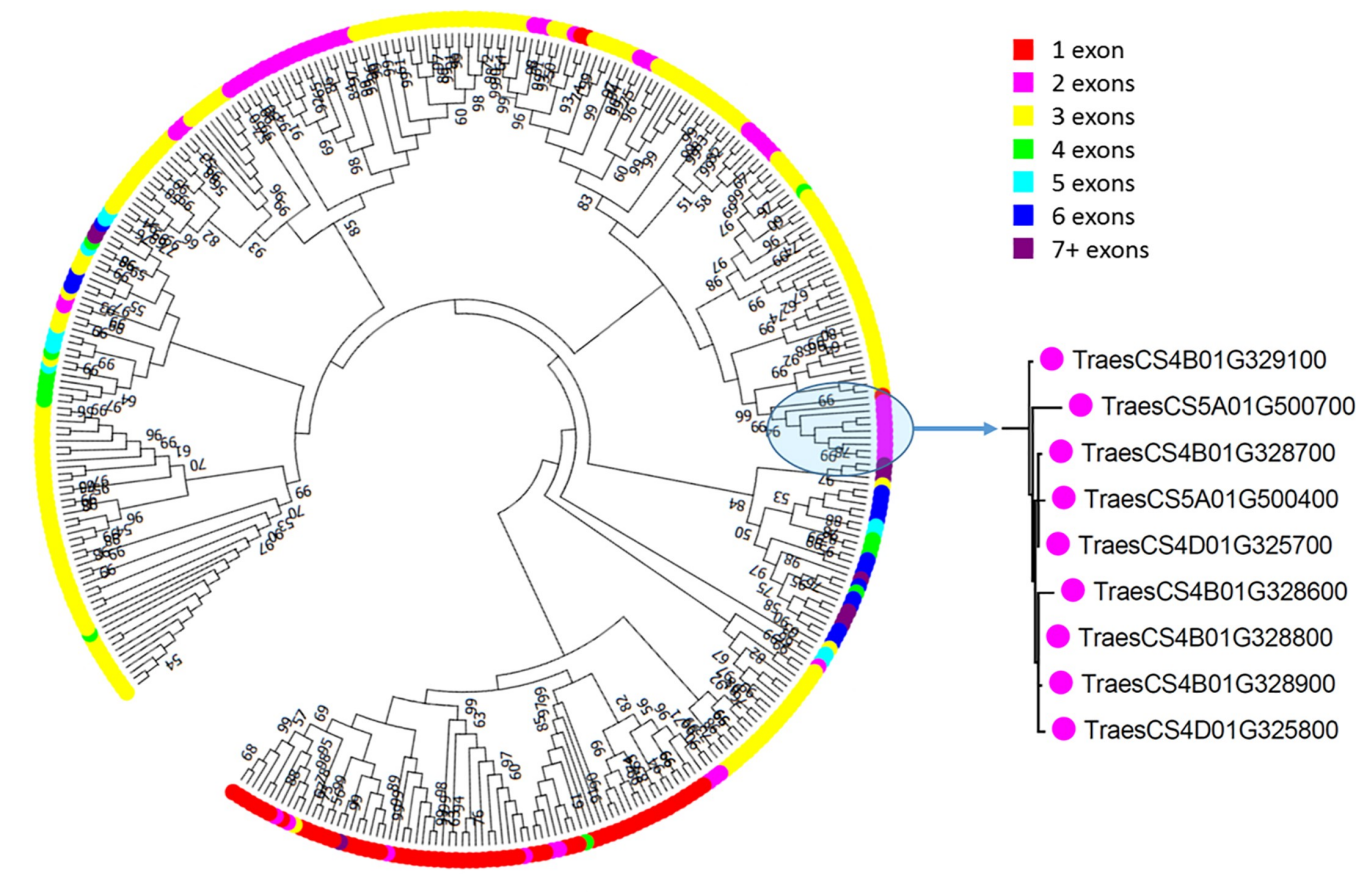
Maximum likelihood phylogeny of the 361 common complete protein sequences. The zoom focused on the clade studied as a case of duplication. Colors indicate the number of exons in each sequence.

The evolution of the TaNAC family was evaluated by studying the phylogenetic relationships between *TaNAC* genes. A phylogenetic tree based on multiple sequence alignment of the common set including 361 full-length proteins was constructed (Fig 1), using the Maximum Likelihood method.

The phylogenetic tree showed that, in general, sequences contained in the clades belong to the same homoeologous groups. Based on sequences alignment, 156 *TaNAC* genes belonging to A, B, and D genomes were grouped into 52 complete homoeologous groups in the phylogenetic tree, representing 43% of the total *TaNAC* genes. Another 56 incomplete homoeologous groups containing only two members were identified and represent 31% of the total *TaNAC* genes. Finally, 93 sequences could not be linked to any homoeologous group. It is interesting to note that several sequences with the same exon-intron structuration and clustered on the wheat genome are grouped together in the phylogenetic tree.

In addition, these clades contain genes originating from non-homoeologous chromosomes. These observations suggest that the expansion of the TaNAC family might be the result of both local and interchromosomal duplication events. To test this hypothesis, we retrieved the genomic sequences of 50 Kb loci spanning *TaNAC* genes and carried out pairwise alignments using the YASS algorithm [54].

**Fig 2:**
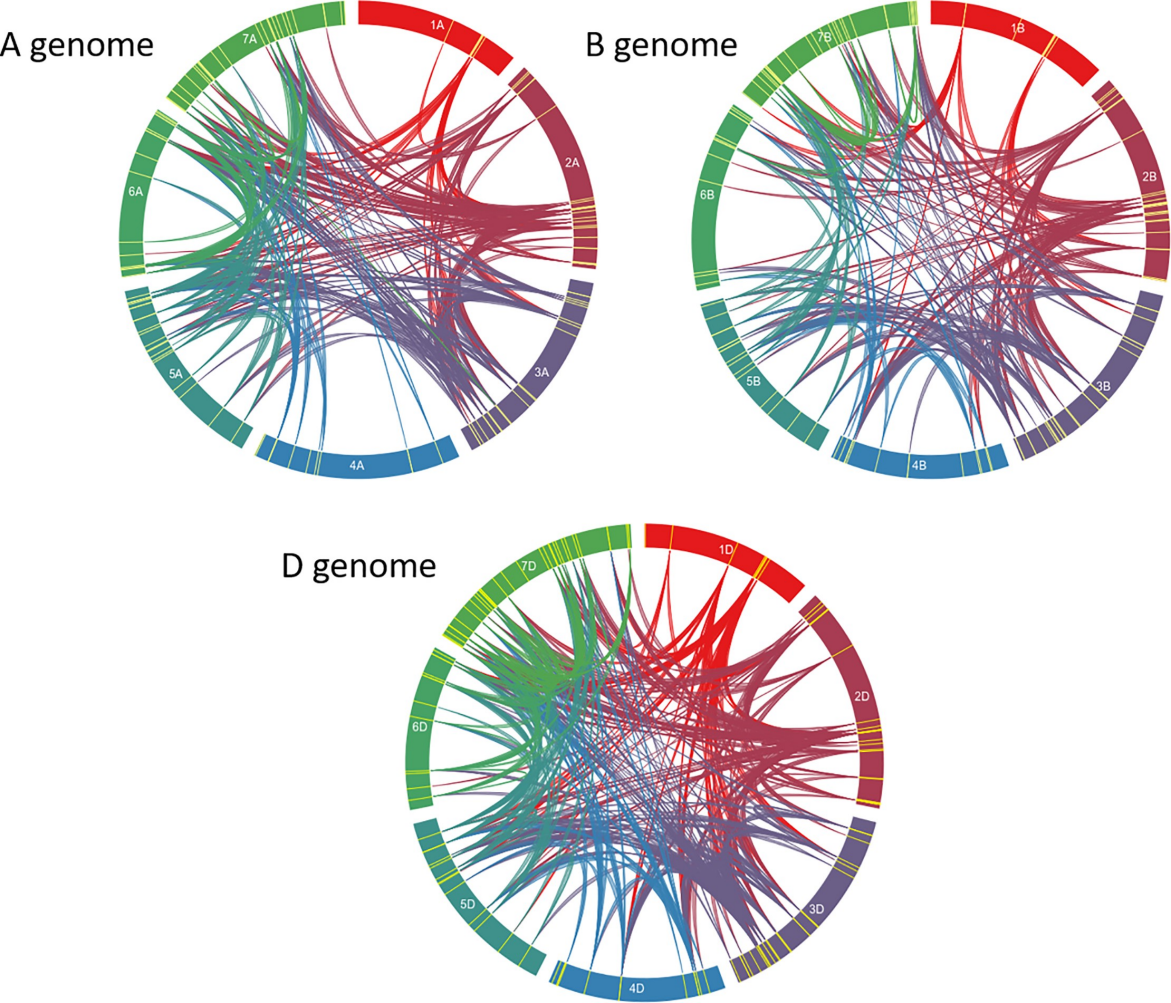
**Circos diagram of the seven chromosomes of genomes A, B, and D.** Yellow features indicate the presence of a TaNAC. Bands link two chromosomal regions with more than 90% of homology and a minimal length of 3 Kb.

For the purpose of simplicity, we first considered duplications within each subgenome. Fragments displaying at least 90% identity over at least 3 Kb were retained. Using these criteria, 318 duplicated fragments were identified within the subgenome A, with mean-length = 5.75 Kb and median-length = 5.255 Kb. The largest fragment was 13.093 Kb. Concerning the subgenome B, 207 duplicated fragments were detected, with mean-length = 5.546 Kb and median-length = 5.146. The largest duplicated fragment was 10.009 Kb. Concerning the subgenome D, 428 duplicated fragments were detected, with mean-length = 5.639 Kb and median-length = 5.332. The largest duplicated fragment was 9.947 Kb (Fig 2, S4 Table).

**Fig 3:**
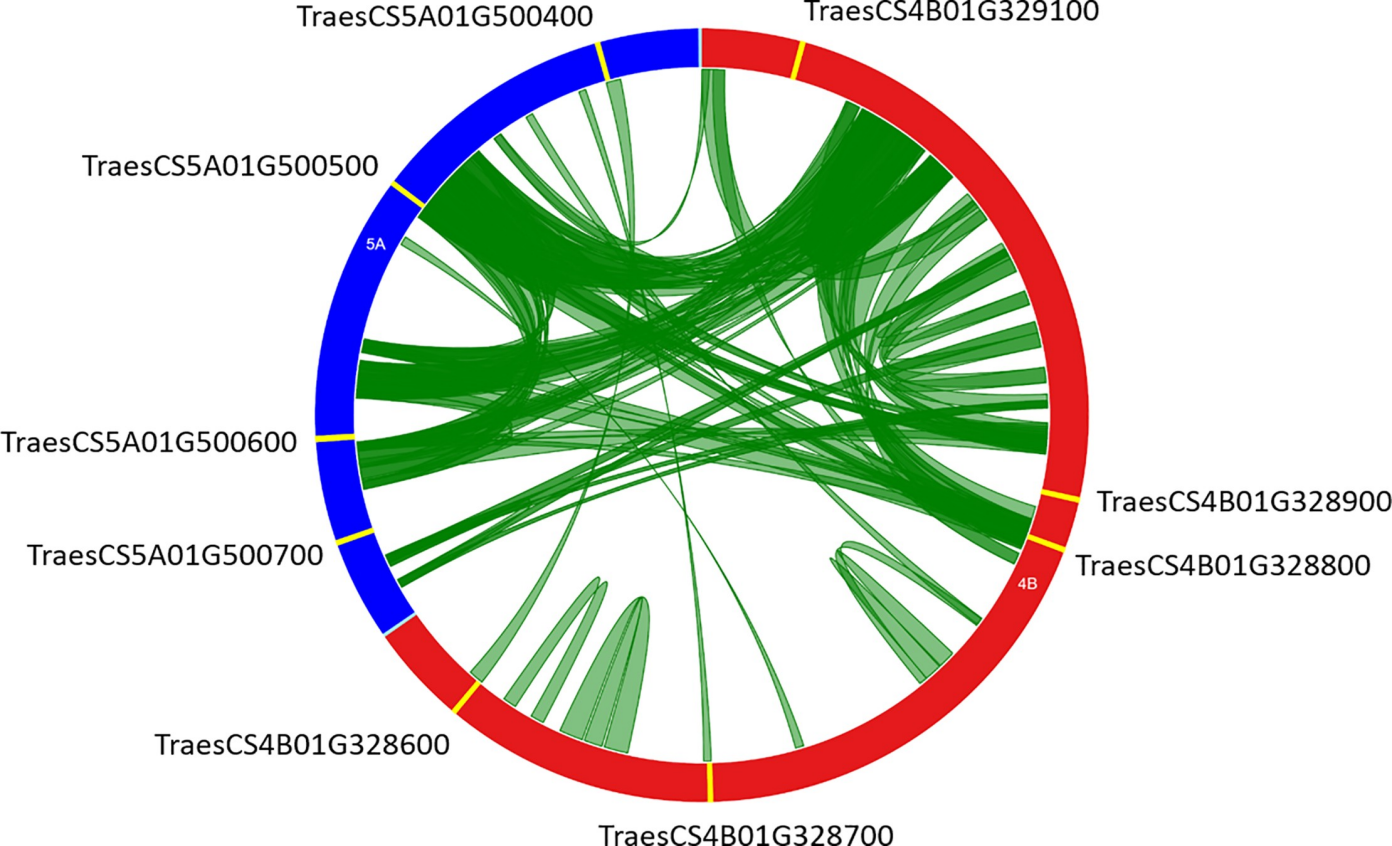
Circos diagram of chromosomes 4B (red, 396.990 Kb) and 5A (blue, 209.278 Kb) genomic regions containing nine *TaNAC* genes (yellow features). Green bands link two chromosomal regions with more than 80% of homology and a minimal length of 2 Kb.

To complete that first analysis and provide also insight into duplications between subgenomes and consequently into the expansion pattern of the TaNAC family, we carefully surveyed one robust clade containing *TaNAC* genes originating from each wheat subgenome. This clade (pointed out in Fig 1 and boxed in blue in S3 Fig) was chosen because it contained several *TaNAC* belonging to A (2 sequences from chromosome 5), B (5 sequences from chromosome 4), and D genomes (2 sequences from chromosome 4). Careful examination of regions containing the nine genes of this clade indicated that *TaNAC* genes from a given chromosome are physically tightly linked (separated by maximum one gene). To avoid homoeology relationships between subgenomes, which may obscure duplications, the subgenome 4D containing two *TaNAC* genes was not further considered. By using a 25 Kb window around the most distal *TaNAC* genes within each locus, we retained a first 396.990 Kb long (619563787 to 619960777 bp) locus from the chromosome 4B containing the five *TaNAC* genes and a second 209.278 Kb long (666499425 to 666708703 bp) locus from the 5A containing four *TaNAC* genes. Over the four NAC sequences found on this chromosome, only TraesCS5A01G500400 and TraesCS5A01G500700 genes were present in the set of 361 sequences and studied for duplication. A dot plot of these two genomic regions (S4 Fig) highlighted numerous duplicated fragments in sense and antisense orientations. To clarify the nature and the extent of duplications, we identified all the duplicated fragments with a minimal length of 2 Kb and at least 80% identity. A total of 119 non-overlapping duplicated fragments were identified, of which 54 fragments correspond to intrachromosomal duplications (31 on chromosome 4B and 23 on 5A). The remaining 65 duplicated fragments correspond to interchromosomal duplications. The majority of these duplications (66.7%) represents a length less than 4 Kb, but a fragment has the maximum size of 9,006 bp. Duplications between loci of these genomic regions are presented in Fig 3. About 41% of these fragments (49) are duplicated in the sense orientation (S5 Table).

**Fig 4:**
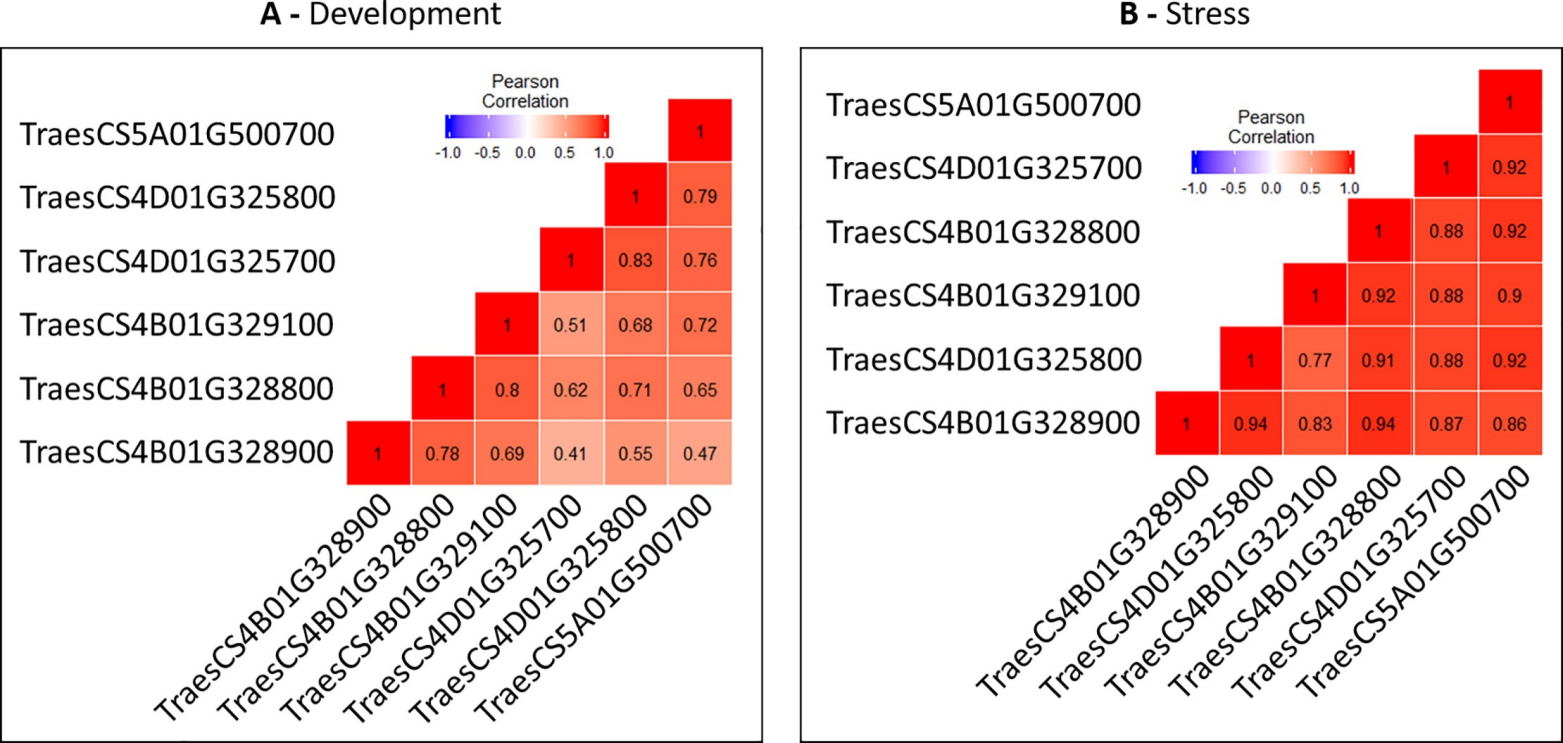
Pearson correlations between duplicated genes of the studied 4B-4D-7B-5A chromosomal regions during development (A) and stress (B).

To test whether some duplicated genes could evolve differently, we compared the normalized expression patterns of duplicated genes in five wheat organs during three developmental stages: root (harvested at seedling, three leaves and meiosis), stem (harvested at spike at 1 cm, two nodes and anthesis), leaf (seedling, three tillers avec 50°Cd after anthesis), spike (harvested at two nodes, meiosis and anthesis), and grain (harvested at 50°Cd, 350°Cd and 700°Cd after anthesis [55]. In parallel, the expression profiles of the same genes were compared in response to abiotic stresses (heat, drought, and combined heat-drought) [56]. Only genes that were expressed in both studies were retained for comparison. The correlation (Pearson coefficient) was computed between pairs of genes (Fig 4).

During development, the Pearson coefficient ranged from 0.41 (between TraesCS4D01G325700 and TraesCS4B01G328900) to 0.83 (between TraesCS4D01G325800 and TraesCS4D01G325700). In contrast, correlations were higher during responses to stress and ranged from 0.77 to 0.94. These results indicate that the members of this duplication display similar expression profiles in response to the abiotic stresses tested but a substantial variation in expression profiles is observed during development between the same members as shown by the behavior of the two paralogs TraesCS4D01G325700 and TraesCS4B01G328900 (Fig 4).

In a similar way, an examination of a second duplication (boxed in red in the phylogenetic tree provided in S3 Fig) was performed. In this case, the expression profiles of the paralogs were highly correlated during development while they could be weakly correlated during responses to abiotic stresses (*i*.*e*. between TraesCS2B01G118500 and TraesCS2A01G101100) (S5 Fig).

### *In silico* analysis of the expression of *TaNAC* genes during development or in response to abiotic stresses

In order to monitor the expression of wheat *TaNAC* genes during development and in response to several abiotic stresses, we took advantage of valuable resources publicly available. In particular, we used a compendium of RNA-seq reads corresponding to various experiments and mapped to the wheat genome by Borrill *et al*. [45]. Raw counts corresponding to five organs at three developmental stages [55] or to abiotic stress treatments [56] were downloaded, normalized using TMM and voom methods, then Differentially Expressed Genes (DEG) were identified using an absolute logFC = 1 and an adjusted p = 0.05 as thresholds (R package limma) [57].

#### Responses of *TaNAC* genes to abiotic stresses

**Fig 5:**
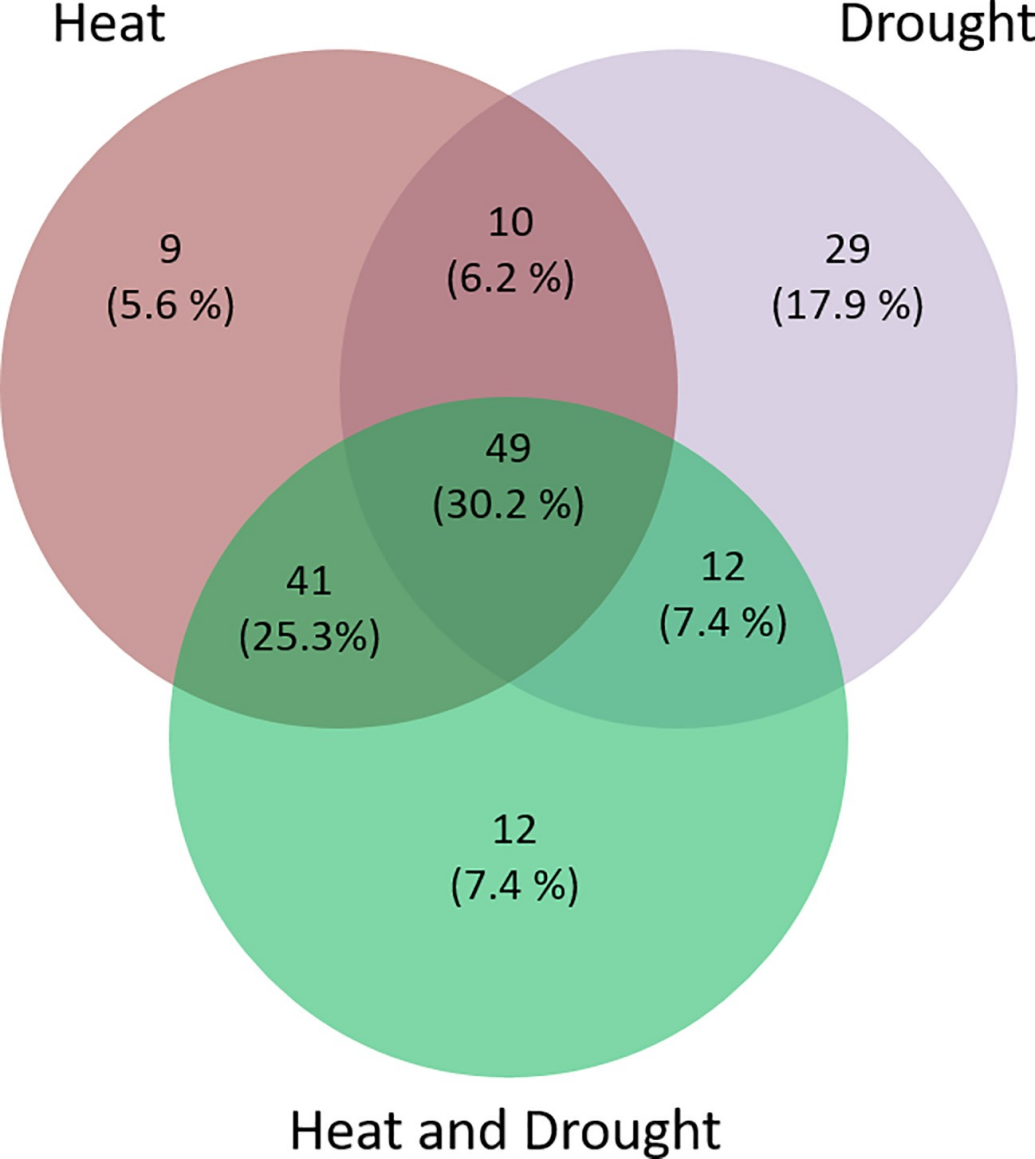
Venn diagram of the 162 differentially expressed *TaNAC* genes in response to heat, drought, and heat + drought stress.

We identified differentially expressed wheat genes in one or several comparisons. The Table 1 shows the details of this analysis where DEG are denoted up- or down-regulated genes in comparison with the non-treated control sample. Among the whole set of DEG genes, 162 were annotated as *TaNAC* genes and dispatched as follows: 109 unique *TaNAC* genes were identified in heat treatments, 100 in drought treatments and 114 in heat-and-drought treatments. Some of them are common in two or more conditions (Fig 5). Table 1 and S6 Table show the distribution of these differentially expressed *TaNAC* genes alongside their logFC and the associated adjusted p-value.

**Table 1:** Number of differentially expressed genes in response to two abiotic stresses.

	Heat stress (40°C) 1h	Heat stress (40°C) 6h	Drought 1h	Drought 6h	Heat + Drought 1h	Heat + Drought 6h
**Number of down-regulated genes**	6763	11420	339	6426	7018	11288
**Number of down-regulated *TaNAC* genes**	24	39	5	28	28	41
**Number of up-regulated genes**	9837	11195	3117	6352	11796	11629
**Number of up-regulated *TaNAC* genes**	43	31	40	54	43	43

#### Expression of *TaNAC* genes during development

**Fig 6:**
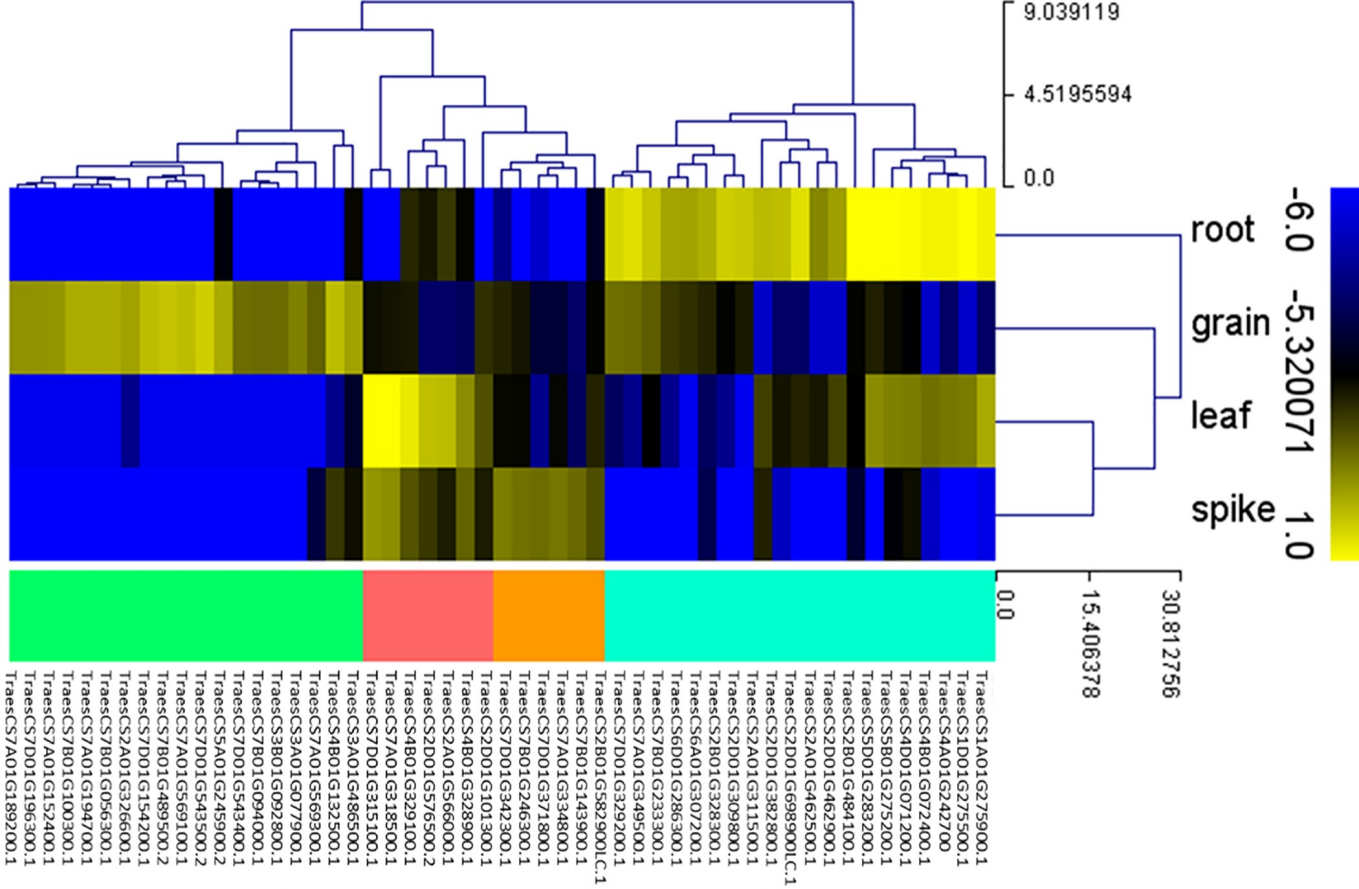
Heat map of 52 *TaNAC* genes expression profile in four organs (grain, spike, leaf, and root), using the RNAseq data of Choulet *et al*. [55]. These 52 *TaNAC* genes have a highly preferred expression pattern distributed as follows: 19 in the grain (green branches), 7 in the leaf (red branches), 20 in the root (blue branches), and 6 in the spike (orange branches). This figure was generated using MeV software. Hierarchical clustering was performed for the transcript abundance from all conditions.

The *TaNAC* genes have been shown to be involved in plant development with expression profiles that can vary between organs and during organ development. In order to detect preferentially expressed *TaNAC* genes in certain wheat organs and at certain stages, we used the RNA-seq data of Choulet *et al*. [55], comprising five organs and three developmental stages per organ. We then also selected the most differentially expressed *TaNAC* genes compared to other tissues using a logFC threshold of +12. Using these relatively stringent criteria, we identified 52 *TaNAC* genes with a highly preferred expression pattern distributed as follows: 19 in the grain, 7 in the leaf, 20 in the root, 6 in the spike and none in the stem (Table 2, Fig 6).

**Table 2:** Number of differentially expressed genes (A) and list of preferentially expressed *TaNAC* genes (B) in five wheat organs.

A	Grain	Leaf	Spike	Stem	Root
Number of down-regulated genes	6505	5815	6533	6356	6362
Number of up-regulated genes	6862	5508	4468	5117	8447
B					
Transcript	Organ with a preferential expression of the gene			logFC	Adjusted P.Value
TraesCS2D01G576500.2	Leaf			18.178	0.0009
TraesCS4B01G329100.1	Leaf			17.573	0.0121
TraesCS2D01G101300.1	Leaf			17.472	0.0231
TraesCS4B01G328900.1	Leaf			17.299	0.0298
TraesCS7A01G318500.1	Leaf			16.701	0.0074
TraesCS7D01G315100.1	Leaf			16.4	0.0356
TraesCS2A01G566000.1	Leaf			16.21	0.0377
TraesCS7B01G489500.2	Grain			24.77	0.0026
TraesCS7D01G543500.2	Grain			24.394	0.007
TraesCS7A01G569100.1	Grain			24.269	0.0038
TraesCS2A01G326600.1	Grain			24.025	0
TraesCS7B01G100300.1	Grain			23.802	0.0025
TraesCS7A01G189200.1	Grain			23.73	0.0002
TraesCS7B01G056300.1	Grain			23.723	0.005
TraesCS7D01G154200.1	Grain			23.719	0.007
TraesCS7A01G194700.1	Grain			23.663	0.0059
TraesCS7D01G196300.1	Grain			23.432	0.0031
TraesCS7B01G094000.1	Grain			23.334	0.0005
TraesCS7A01G152400.1	Grain			23.258	0.0009
TraesCS3B01G092800.1	Grain			23.077	0.0007
TraesCS4B01G132500.1	Grain			22.886	0
TraesCS7D01G543400.1	Grain			22.879	0.0004
TraesCS5A01G245900.2	Grain			22.67	0.0147
TraesCS7A01G569300.1	Grain			21.729	0.0011
TraesCS3A01G486500.1	Grain			20.566	0
TraesCS3A01G077900.1	Grain			20.52	0.0329
TraesCS2D01G309800.1	Root			23.596	0
TraesCS2A01G462500.1	Root			23.17	0
TraesCS2A01G311500.1	Root			22.266	0
TraesCS1D01G275500.1	Root			21.745	0
TraesCS2D01G462900.1	Root			21.084	0.0002
TraesCS6A01G307200.1	Root			20.627	0
TraesCS2B01G328300.1	Root			20.483	0
TraesCS2B01G484100.1	Root			20.365	0.0002
TraesCS6D01G286300.1	Root			20.325	0
TraesCS2D01G698900LC.1	Root			19.923	0
TraesCS7D01G329200.1	Root			19.261	0.0007
TraesCS7B01G233300.1	Root			19.259	0.0001
TraesCS7A01G349500.1	Root			19.001	0.0009
TraesCS1A01G275900.1	Root			18.528	0.0001
TraesCS4A01G242700	Root			18.32	0.0008
TraesCS4B01G072400.1	Root			18.291	0.0003
TraesCS5B01G275200.1	Root			18.04	0.0021
TraesCS5D01G283200.1	Root			17.985	0.0045
TraesCS2D01G382800.1	Root			17.401	0.0022
TraesCS4D01G071200.1	Root			16.877	0.0136
TraesCS7D01G371800.1	Spike			19.526	0.0002
TraesCS7B01G143900.1	Spike			18.488	0.0025
TraesCS7A01G334800.1	Spike			18.372	0.0042
TraesCS7B01G246300.1	Spike			18.219	0.0053
TraesCS2B01G582900LC.1	Spike			17.862	0.005
TraesCS7D01G342300.1	Spike			17.198	0.0151

A *TaNAC* gene is preferentially expressed in a tissue if i) its logFC is the highest in this tissue vs the others and ii) the logFC threshold is ≥ 12.

### *TaNAC* expression analysis in field under drought stress

**Fig 7:**
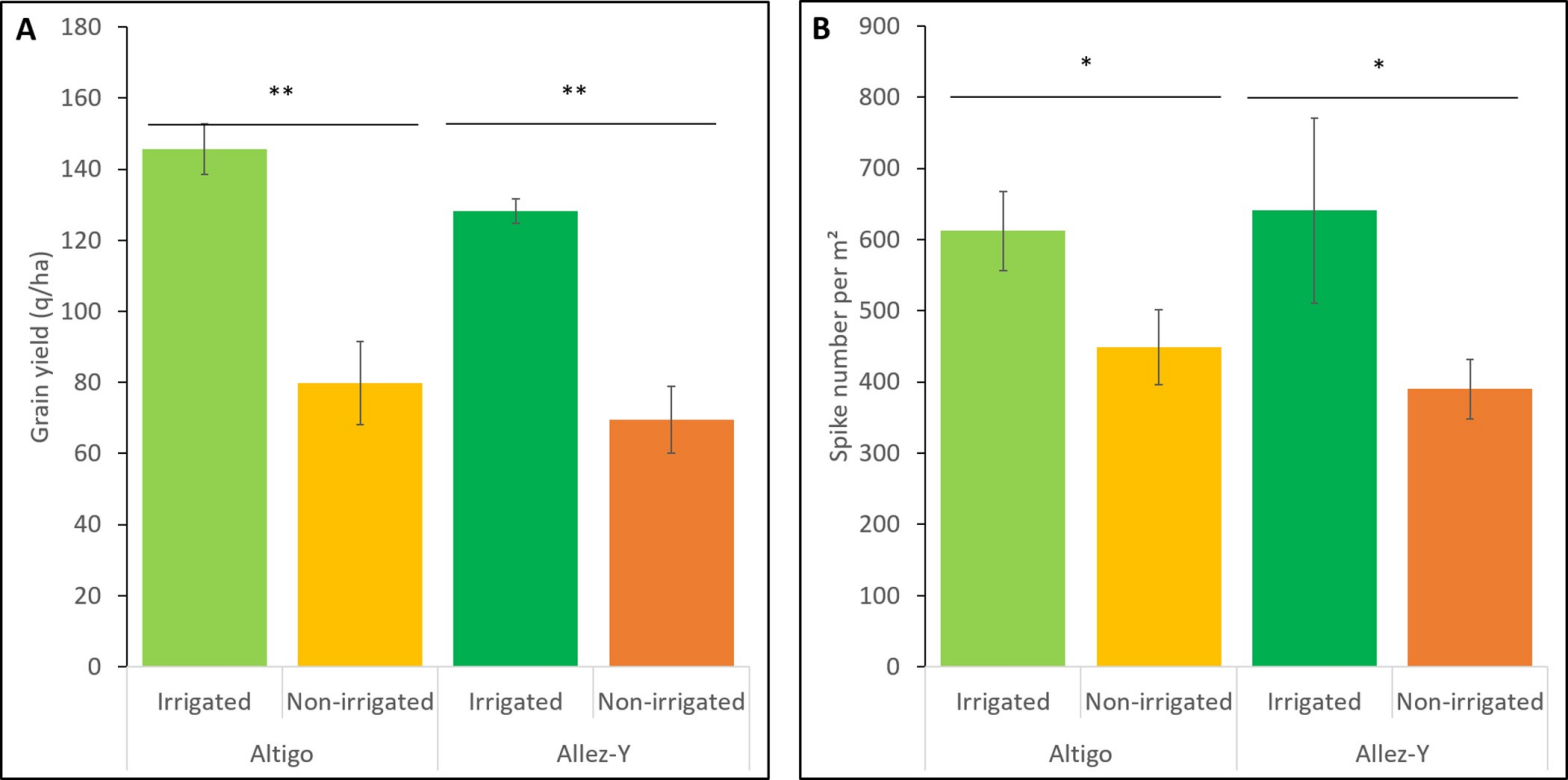
**Drought effect on the grain yield (q/ha) (A) and spike number per m**^2^ **(B) of two genotypes (Altigo and Allez-y).** Mean values and standard deviation were obtained from three replicates (Student’s T test, * indicates a P < 0.05 and ** indicates a P < 0.01).

Grain yield and spike number per m^2^ were used to characterize the effect of the water stress generated in the drought trial. Under non-irrigated treatment, yield losses of 45.2% and 45.8% were observed for Altigo and Allez-y genotypes respectively. In the same way, a decrease of the spike number per m^2^ was observed (-26.6% for Altigo and -39.1% for Allez-y) (Fig 7). which translates to a high level of water stress that affected plant growth [60].

#### *TaNAC* gene expression profile under irrigated conditions

The *in silico* analysis resulted in a set of *TaNAC* genes tagged as DEG. A subset of 23 *TaNAC* displaying high fold changes in their expression were selected and analyzed in plants grown in the field under agronomic conditions. Their expression was monitored in two organs (leaf and grain) at two stages representative of grain development: cell division (220°Cd) and grain filling (450°Cd) in Altigo and Allez-y.

**Fig 8:**
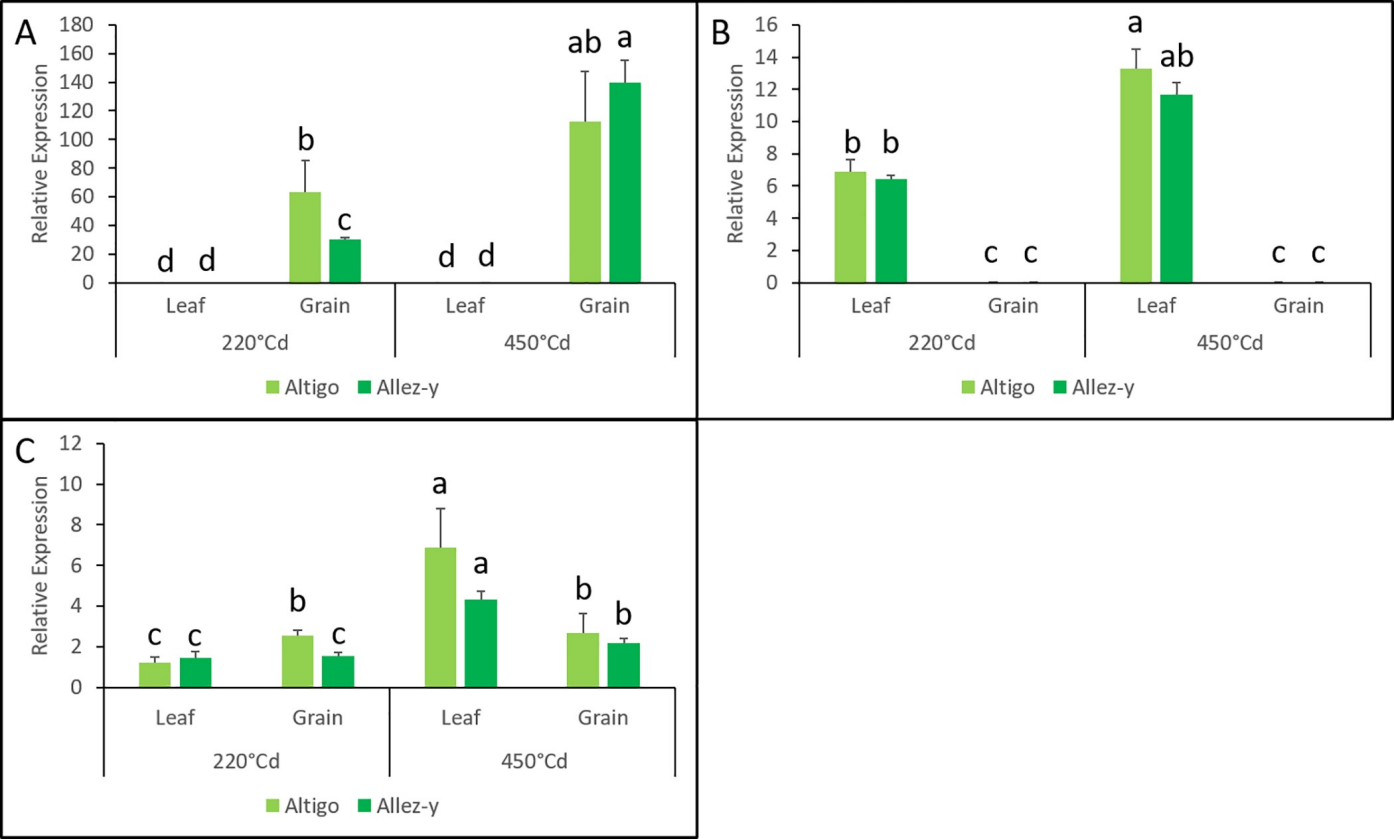
**RT-qPCR analysis of three *TaNAC* genes (TraesCS5A01G245900.2 (A), TraesCS7D01G315100.1 (B), and TraesCS2A01G101100.1 (C)) in leaves and grains at 220°Cd and 450°Cd in irrigated conditions.** Mean values and standard deviation were obtained from three replicates. According to a Kruskal-Wallis test, the same letter indicates no significant difference.

The 23 *TaNAC* genes tested in irrigated fields exhibited statistically differential transcript levels between organs (leaves and grains), regardless of the genotype or the stage. Seven genes appeared to be preferentially expressed in the grain and 16 in the leaf. The gene TraesCS5A01G245900 showed the highest expression in the grain compared to the leaf (10 018 times more) whereas TraesCS7D01G315100 was the most expressed gene in the leaf (332 times more than in the grain) (Fig 8A and 8B).

In addition, some *TaNAC* gene expressions varied depending on the developmental stage (220°Cd vs 450°Cd). One gene (TraesCS2A01G566300.1) appeared to be preferentially expressed at 220°Cd while 15 genes were preferentially expressed at 450°Cd. For example, the expression level of TraesCS2A01G101100 gene was found to be 4.19 times more in leaves at 450°Cd compared to 220°Cd (Fig 8C).

#### Expression of *TaNAC* genes under drought stress

The expression data of the 23 selected genes in drought field conditions were presented in a principal component analysis (S6 Fig). The first two Principal Components (PC1 and PC2) explained respectively 83.5% and 10.8% of the total variation. Cumulatively, these two PCs contributed to 94.3% of the total variation of gene expression in response to drought, organ, genotype, and stage conditions. The first axis is explained by gene expression in the leaves under drought conditions at the later stage, 450°Cd (S6 Fig). The second axis is mainly explained by an organ x stage effect. Indeed, grain samples at 450°Cd were plotted in the lowest part of the S6 Fig. In the grain subset, the samples corresponding to early and late grain developmental stages (full form– 220°Cd and empty form– 450°Cd) constitute distinct groups along the second axis (PC2). In addition, considering only the data from grain at 450°Cd, it can be noticed that the two genotypes are plotted into two different groups.

**Fig 9:**
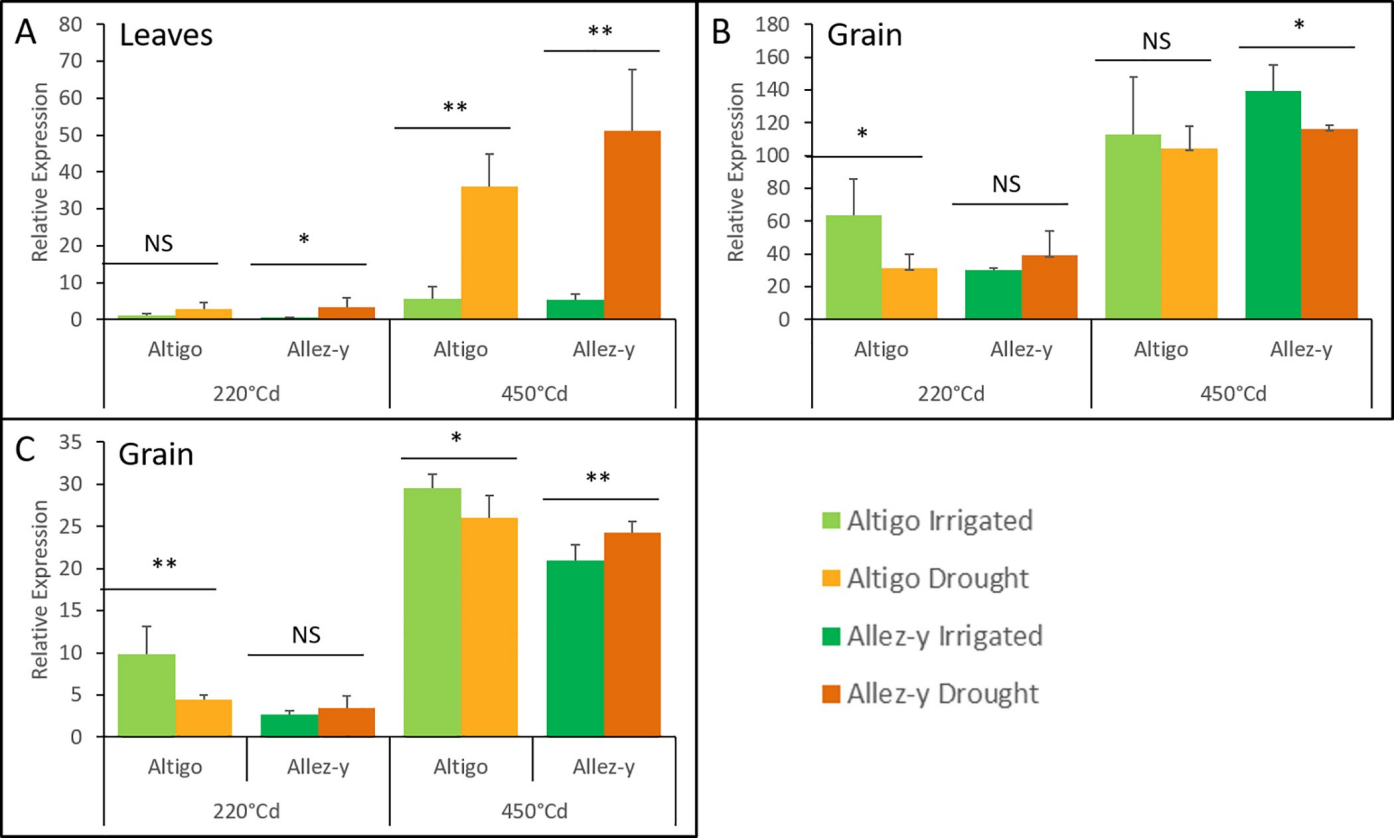
**RT-qPCR expression analysis of TraesCS5A01G143200.1 (A), TraesCS5A01G245900.2 (B), and TraesCS7A01G569100.1 (C).** The 2^-ΔΔCt^ method was used to calculate the ratio of their relative expression levels in drought conditions with the one in irrigated conditions. Mean values and standard deviation were obtained from three replicates (Student’s T test, * indicates a P < 0.05 and ** indicates a P < 0.01).

In leaves, the highest difference under drought stress was observed at 450°Cd compared to 220°Cd, regardless of the genotype (Fig 9A). A genotype effect was observed in response to drought only in grains compared to leaves. More precisely, grain-specific genes were differentially expressed at a different stage depending on the genotype. Indeed, a down-regulation under drought treatment was observed at 220°Cd in Altigo for five grain-specific genes. In Allez-y, four of these transcripts (TraesCS3A01G077900.1, TraesCS5A01G245900.2, TraesCS7B01G056300.1 and TraesCS7B01G100300.1) were down-regulated and the last one was up-regulated (TraesCS7A01G569100 .1) only at 450°Cd (Fig 9B, Fig 9C).

## Discussion

The sequence availability of the whole bread wheat genome and expression data among different wheat genotypes, organs, and conditions are rich resources for systematic analysis of gene transcriptional regulation. Particularly, these data provide useful information for the identification of candidate genes involved in wheat development and its stress response.

Among transcription factors, the NAC family is one of the largest families and they are studied in a plethora of cereals [18,20,21] particularly in response to abiotic stresses [42,64,65], but only scarce information is available on bread wheat.

However, valuable resources and studies have recently been released. Particularly, Borrill *et al*. [45] identified 453 high confidence *TaNAC* retrieved from TGAC database [48]. In addition, large RNA-seq expression data exist on several wheat genotypes, in different organs [55] and abiotic stresses (heat shock or drought) [56] and are available to the scientific community. Based on these data, we studied the organisation of the NAC family in bread wheat and its expansion that could have occurred during evolution through potential duplication events. These resources were also used to identify differentially expressed *TaNAC* genes in wheat grown under drought conditions in field and to validate the potential specialization of some TaNAC members.

### *TaNAC* genes: A large family

First, we identified 488 members of the NAC family in bread wheat using the IWGS RefSeq v1.0 database [47], whereas only 117 members were identified in Arabidopsis, 151 in rice [17] or 168 in durum wheat [21]. This high number is due to the presence of three subgenomes in bread wheat. After improving our dataset with TGAC sequences retrieved by Borrill *et al*. [45], we ended up with 361 sequences representing a high quality consensus subset. Considering the hexaploidy of wheat, one copy of each gene is expected to be carried by each of the three genomes (A, B, and D) creating homoeologous groups among the consensus subset. In this family, 43% of the TaNAC members can be gathered in complete homoeologous groups, 31% form incomplete homoeologous groups and 26% are not linked to a group. A divergence of sequences due to several mutations or a lack of sequences present in the subset could explain the incomplete homoeologous groups. This result has already been observed in other TF families, such as the bZIP family [66].

Similar to what has been described in other plant species such as maize [67] and rice [17], the *TaNAC* gene distribution among the chromosomes was uneven. On the contrary, the three subgenomes carry the same proportion of *TaNAC* genes, which is consistent with the distribution observed in durum wheat, a diploid species [21]. This result suggests that *TaNAC* family expansion was likely prior to a polyploidization event in bread wheat.

### Expansion of the wheat *TaNAC* genes family due to small-scale duplications

Several recent studies have proposed different mechanisms to explain the duplication events and gene expansion in plants. These proposals included, for instance, retrotransposition, duplicated DNA transposition, unequal crossing-over, and polyploidization [68–70]. In addition to these mechanisms, Wicker *et al*. [71] then Glover *et al*. [72] proposed another mechanism leading to gene movement and expansion. In particular, Glover *et al*. [72] showed that massive small-scale interchromosomal gene duplications occurred in the bread wheat genome after double-strand DNA break repair. Consequently, many non-syntenic genes have high sequence similarity. Regardless of the mechanisms involved in gene duplication, this may eventually lead to a pseudogenization, a conservation of the gene function, a subfunctionalization or a neofunctionalization of duplicate genes [73].

In this study, we noticed that several clades in the phylogenetic tree contained sequences that were either physically tightly linked on the same chromosome or mapped to a non-homoeologous group, suggesting that both intra and inter-chromosomal duplication events occurred. To test the possibility of small-scale duplications, we focused on three genomic regions on the chromosomes 4B, 4D, and 5A that encompass 11 *TaNAC* genes, which display highly similar sequences and similar genomic structures (2 exons). By aligning the sequences at the vicinity of these genes, we uncovered a high homology between the genomic regions (Fig 3). This finding support the hypothesis evoked by Glover *et al*. [72] about the expansion of the multi-genes family that is partially due to small-scale duplication driven by repetitive sequences.

### Expansion of the wheat *TaNAC* genes family due to retroposition

Of the 361 high-quality sequences, 68 *TaNAC* genes (19%) are intronless, which is a characteristic of retroposons [74]. The subfamily NAC-h includes 65 of these 68 genes that are grouped in the same clade of the phylogenetic tree. However, in addition to an absence of intron, the presence of a poly(A) tract at the 3’end and a TSD (Target Site Duplication) in a gene sequence constitutes a hallmark of a retroposition event [74]. However, we failed to identify any of these signatures in these sequences. These signatures were possibly obscured by multiple mutations during evolution to such extent that they are no longer recognisable. Furthermore, several intronless *TaNAC* genes of this clade are grouped into homoeologous groups suggesting that retroposition/duplication events probably occurred prior to wheat polyploidization.

Interestingly, the distribution of *TaNAC* intronless genes among chromosomes was found not to be equivalent to the one observed for the whole TaNAC family. For example, although the gene proportions of the whole TaNAC family on the chromosome 3 and 5 are close (13 and 16% respectively), a higher proportion of intronless *TaNAC* genes was noticed on chromosome 3 (29% of the total intronless genes) whereas no intronless genes were observed on chromosome 5.

Taken together, these results indicate that both small-scale duplication and retroposition phenomena led to the expansion of this family of genes. Furthermore, these mechanisms could act in combination, *i*.*e*. a gene retroposition could be followed by small-scale duplications driven by repetitive sequences at the very vicinity of the gene.

For example, TraesCS2A01G328100, TraesCS2A01G328200, TraesCS2A01G328300 and TraesCS2A01G328500 are intronless and tightly linked genes, and could be an example of the retroposition/duplication events that could have occurred during evolution.

### Expression of *TaNAC* genes under drought field conditions

As a major widely spread environmental stress, drought is known to significantly affect wheat yields worldwide. Interactions between plant development and stress response are complex, especially in the field, where crops are submitted to a combination of environmental variables (such as water availability and temperature). Although a few NAC TFs have been studied in wheat compared with the model plants Arabidopsis and rice, it has been shown that they may also be involved in response to environmental conditions [5]. Some *TaNAC* genes have already been studied in bread wheat. For example, Arabidopsis overexpressing *TaNAC2*, *TaNAC29*, or *TaNAC67* display improved tolerance to multiple abiotic stresses [16,42,75]. In the same way, *TaNAC2a*, *TaNAC2D*, *TaNAC4a*, *TaNAC6*, *TaNAC7*, *TaNAC13*, and *TaNTL5* were induced by water stress, high salinity, and/or low temperature, at different intensities [76,77]. Recently, *TaRNAC1* was found to enhance drought tolerance leading to higher biomass, grain yield, and root length [78]. In addition, transgenic wheat overexpressing *TaNAC47* showed a drought-tolerant phenotype that may be induced in response to ABA [79].

Here, we have studied 23 *TaNAC* genes that we identified as the most up-regulated (p-value < 0.05) in specific organs (leaf or grain) or in response to treatments (drought and heat) (S7 Table). Among the 23 selected genes, 20 belong to the subfamilies NAC-a to d, which have been described as Stress Related NAC containing subfamilies [80] (S3 Table).

#### Some *TaNAC* genes exhibit an extreme level of transcript abundance specifically depending on the organ

*NAC* genes are recognized to be largely involved in many developmental and growth processes, such as the differentiation of major organs [81]. Our study highlighted genes that are preferentially expressed in the leaf (16) or in the grain (7) regardless of the genotype, the grain developmental stage, or the field conditions.

Interestingly, of those genes, seven leaf-specific and five grain-specific genes were found, with an expression level at least 10 times higher than in the other organ (S7 Table), suggesting their specific implication in the leaf or grain development. For example, TraesCS7A01G305200.1 gene up-regulated in leaves either in the earliest grain developmental stage (220°Cd) and the grain filling stage (450°Cd), was found to be similar to TaNAC1/TaNACS already described by Zhao *et al*. [37]. In this work, the authors highlighted an increase in nitrogen concentration in wheat grains from lines over-expressing the TaNAC-S protein (TaNAC1) and a stay-green phenotype. These authors concluded that NAC TFs, expressed mainly in the leaves, may participate to delay post-anthesis foliar senescence (stay-green phenotype) through a better remobilization of the nutrients during senescence. Another gene responding mainly in leaf in our experiment (TraesCS4D01G176000.1) is homologous to a *NAC* gene in the rose, *RhNAC100*, that has been characterized as a repressor of cellular expansion of rose petals [82], suggesting its involvement in the determination of cell size.

Among the seven up-regulated *TaNAC* grain-specific genes, one gene (TraesCS7A01G569100.1) was found to be homologous to ONAC20 and ONAC026 described in rice [24]. In this latter study, the authors showed that three *NAC* genes (ONAC020, ONAC026 and ONAC023) are expressed specifically during seed development at a very high level, suggesting a potential role of these genes, *via* their heteromerization capability, in the regulation of the grain size/weight. Another gene, TraesCS3A01G077900.1, was found to be homologous to a barley *HvNAC019* gene which is preferentially expressed in grain, indicating that this gene could regulate seed development in association with two other genes (*HvNAC017* and *HvNAC018*) [83].

#### Leaf-specific *TaNAC* genes are up-regulated at the grain filling stage in response to drought

Sixteen genes were found differentially expressed in both leaves and grains between the early grain developmental stage (cell division) and the grain filling stage, and mainly up-regulated at 450°Cd (15 genes).

Drought stress during grain filling stimulates leaf senescence and enhances remobilization of water-soluble carbohydrates to the grains [84]. Interestingly, we found a set of eight *TaNAC* genes that were preferentially expressed in leaves during grain filling in response to drought. This finding may indicate that these genes could participate in the resource remobilization from the leaves to the grains. For example, the three members of the same homoeologous group, located on chromosome 5 (TraesCS5A01G143200.1 (Fig 9A), TraesCS5B01G142100.1 and TraesCS5D01G148800.1) were found similar to *TaNAC69*. This gene has been well-characterized as a drought stress regulator [85], and was shown to improve grain yield. However, its expression was found mainly in roots compared to leaves [65,85,86]. It is worthy to note that, in our study, the genome A copy-gene was expressed at a higher level than the two other homoeologous copies in leaves. This finding may suggest that the B and D copies could be related to the root-specific expression that has not been measured in this study. The homologous gene in rice (*OsNAC10*) has also been demonstrated to be involved in drought adaptation mainly by enhancing the level of stress-protection genes, such as chitinase, ZIM family gene, and glyoxalase I family gene; and increases grain yield significantly under field drought conditions [87]. Also, two members of the same homoeologous group (TraesCS4D01G176000, TraesCS4B01G174000) are homologs to the rice gene *ONAC39* (*OSCUC1/ONAC039*) that has also been described by Jeong *et al*. [87] as a drought responsive gene (fold change: 9.44). The genes of this homoeologous group in wheat may have similar functions and indicates that these genes may be associated with drought stress.

Another wheat gene (TraesCS2A01G101100, Fig 8C) was up-regulated in leaves under drought at 450°Cd in our field conditions. In contrast, its homolog, *TaGRAB2*, which was involved in response to biotic stress (a plant DNA virus *Geminiviridae*), showed an expression pattern barely detectable in leaves [39]. These findings suggest that the same gene could be expressed or not in leaves, depending on the stress.

Finally, no characterized homolog has been found in the literature for TraesCS3B01G303800.1 and TraesCS6D01G390200.1. These genes may be interesting candidates for field drought tolerance and require further investigations.

#### Grain-specific *TaNAC* genes respond differentially to drought according to the genotype

Five *TaNAC* genes (TraesCS3A01G077900.1, TraesCS5A01G245900.2, TraesCS7A01G569100.1, TraesCS7B01G056300.1, and TraesCS7B01G100300.1) are grain-specific. Interestingly, their behavior in response to drought varies according to the genotype. They are down-regulated under drought treatment during the early grain developmental stage (cell division) in the genotype which exhibited the highest number of spikes/m^2^ (Fig 7B) (Altigo); whereas they are down-regulated (4 genes) or up-regulated later during the grain filling stage (TraesCS7A01G569100.1), in the other genotype (Allez-y) (Fig 9B, Fig 9C). These results suggest that, depending on the genetic background, those genes may harbor different alleles that could affect their drought tolerance.

Four of these five genes (TraesCS7A01G569100.1, TraesCS5A01G245900.2, TraesCS7B01G056300.1, and TraesCS7B01G100300.1) have been found previously differentially expressed in grain in response to a moderate warming of +8°C applied during the grain cell division stage [88]. These results suggest that these four *TaNAC* genes may be involved in response to close abiotic stresses (drought and high temperature), such as other *NAC* genes like *MlNAC9*, *SlNAC3*, *ONAC022*, *GmNAC3* and *GmNAC4*, that were described involved in several environmental stresses [19,89–91].

## Conclusion

In conclusion, we performed a comprehensive study of NAC TFs in the bread wheat genome (*T*. *aestivum*), cultivar *Chinese Spring*. We identified a high quality set of 361 *TaNAC* genes in common with those described in Borrill *et al*. [45]. We provided evidences as to how small scale duplications and retroposition contributed to the expansion of this family of genes. Finally, the expression patterns of 23 *TaNAC* genes in two organs at two stages under drought in the field, combined with our study of duplication events, revealed a functional diversification of this gene family’s members. Further functional characterizations of five grain specific *TaNAC* genes are in process to identify their role in the wheat grain development and in abiotic stress responses.

## Supporting information

S1 Fig Chromosomal distribution of the *TaNAC* gene family in bread wheat. Using their chromosomal location information, 451 sequences have been mapped on the seven chromosomes of the three wheat subgenomes A (red), B (green), and D (blue). The first seven letters of the sequence names have been removed (*e*.*g*. 4B01G328600 refers to TraesCS4B01G328600, located on the chromosome 4B according to the IWGSC annotation). The position of each *TaNAC* can be estimated using the scale on the left. The two circles show the chromosomal regions studied as an example of duplication.
(TIF)

S2 Fig Exon number in the genomic sequence of the 488 TaNAC members. The percentage of sequences is indicated at the top of each histogram.
(TIF)

S3 Fig Maximum likelihood phylogeny of the 361 consensus sequences using complete protein sequences. The blue and red boxes focused on the clades studied as case of duplication. The number of exons is represented by a red (1 exon), pink (2 exons), yellow (3 exons), green (4 exons), clear blue (5 exons), dark blue (6 exons), or violet (7 and more exons) circle.
(PDF)

S4 Fig Structural conservation between the two regions of chromosomes 4B and 5A genomics sequences, revealed by dot plot analysis. Each gene location is indicated on the x- and y-axis by a blue arrow. Green and red dots indicate sense and antisense homologies respectively.
(TIF)

S5 Fig Pearson correlations between duplicated genes of the studied 2A-2B-2D chromosomal regions during development (A) and stress (B).
(TIF)

S6 Fig Biplot showing the first two variables of the principal component analysis (PC1 and PC2). Expression of the 23 *TaNAC* genes in leaves and grains of Altigo (square) and Allez-y (circle) genotypes at 220°Cd (full form) and 450°Cd (empty form) in irrigated (blue) and drought (red) conditions.
(TIF)

S1 Table Blast results using Blast2Go software [92] on 488 TaNAC sequences. (XLSX)

S2 Table List of primers used in this study. (XLSX)

S3 Table TaNAC sequences and structural information extracted from the IWGS RefSeq v1.0 database and their TGAC correspondence. (XLSX)

S4 Table Duplicated fragments contained within the three genomes of the bread wheat, with a minimal length of 3 Kb and at least 90% of identity. (XLSX)

S5 Table Duplicated fragments contained between genomic regions of 4B and 5A chromosomes of the bread wheat, with a minimal length of 2 Kb and at least 80% of identity. (XLSX)

S6 Table Distribution of the differentially expressed *TaNAC* genes in response to heat and drought stresses, alongside their logFC and the associated adjusted p-value. (XLSX)

S7 Table Specific expression of 23 *TaNAC* genes studied by RT-qPCR and their orthologues in other species [24, 37, 39, 40, 65, 82, 83, 85, 87, 93–96]. The preferential expression have been verified by a Student Test, with an e-value threshold at 0.05.
(XLSX)
