# Supplementary figures and images for: Genome-wide analysis, expansion and expression of the NAC family under drought and heat stresses in bread wheat (T. aestivum L.)
Source: PLoS One. 2019 Mar 6;14(3):e0213390. doi: 10.1371/journal.pone.0213390 (PMC6402696; doi:10.1371/journal.pone.0213390)

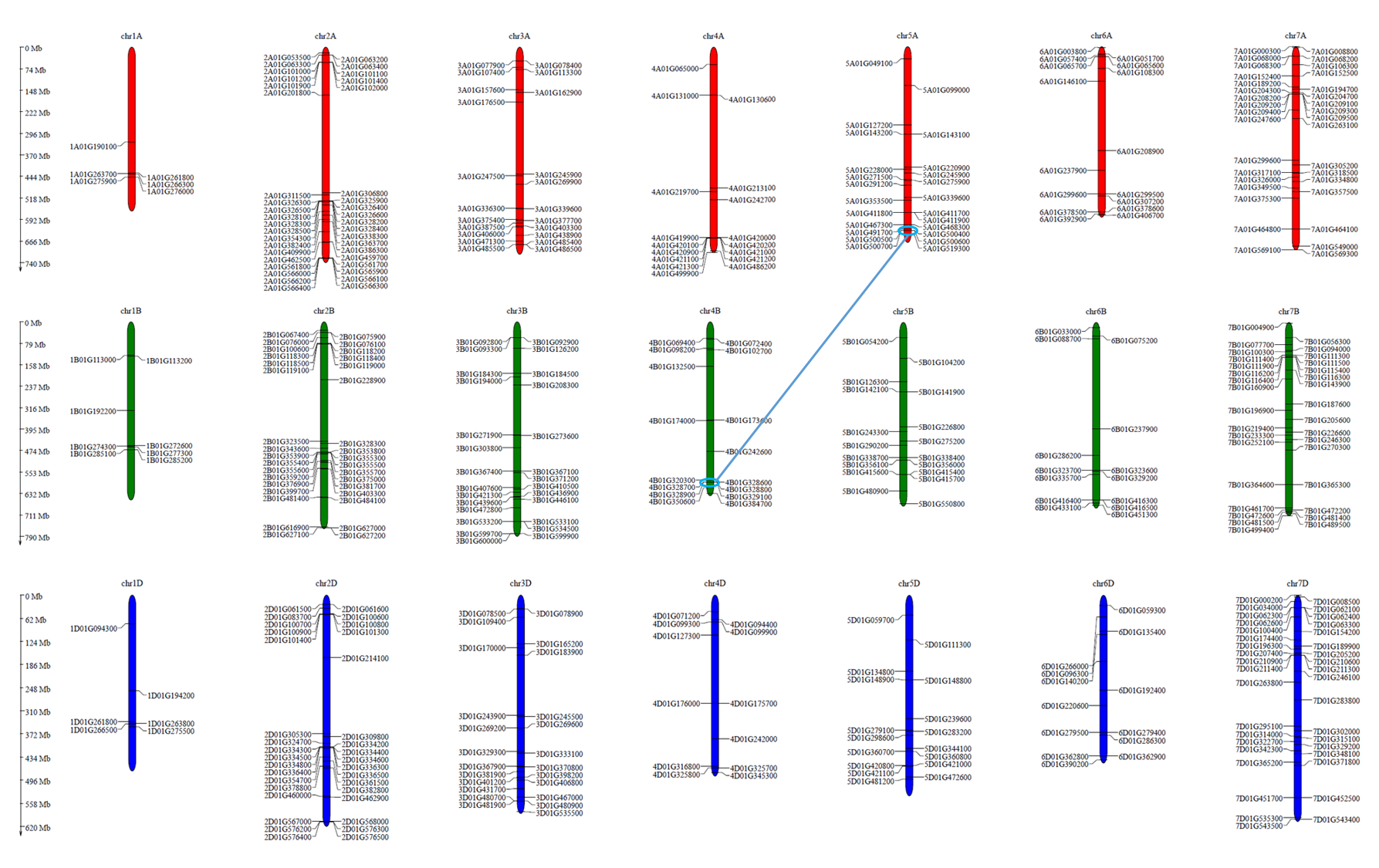

Supplement: S1 Fig — Using their chromosomal location information, 451 sequences have been mapped on the seven chromosomes of the three wheat subgenomes A (red), B (green), and D (blue). The first seven letters of the sequence names have been removed (e.g. 4B01G328600 refers to TraesCS4B01G328600, located on the chromosome 4B according to the IWGSC annotation). The position of each TaNAC can be estimated using the scale on the left. The two circles show the chromosomal regions studied as an example of duplication. (TIF) [file pone.0213390.s001.tif]

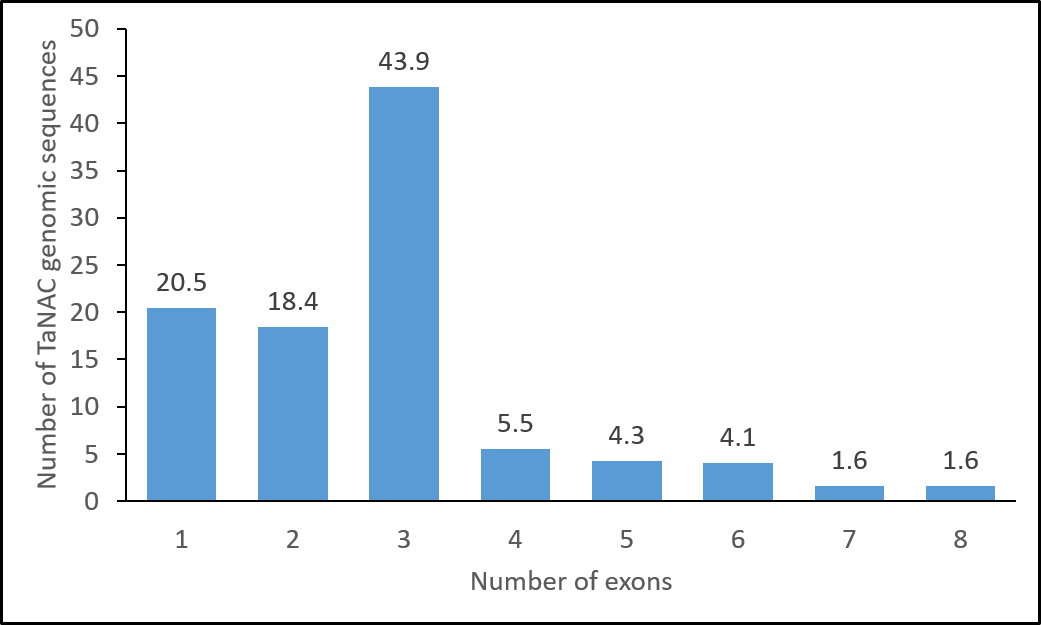

Supplement: S2 Fig — The percentage of sequences is indicated at the top of each histogram. (TIF) [file pone.0213390.s002.tif]

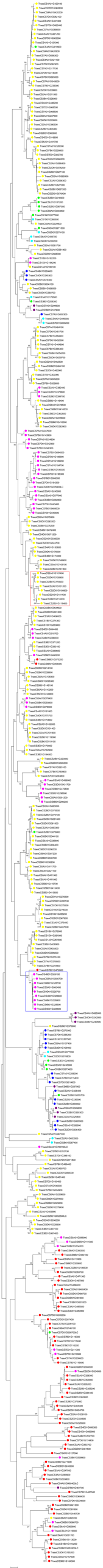

Supplement: S3 Fig — The blue and red boxes focused on the clades studied as case of duplication. The number of exons is represented by a red (1 exon), pink (2 exons), yellow (3 exons), green (4 exons), clear blue (5 exons), dark blue (6 exons), or violet (7 and more exons) circle. (PDF) [file pone.0213390.s003.pdf]

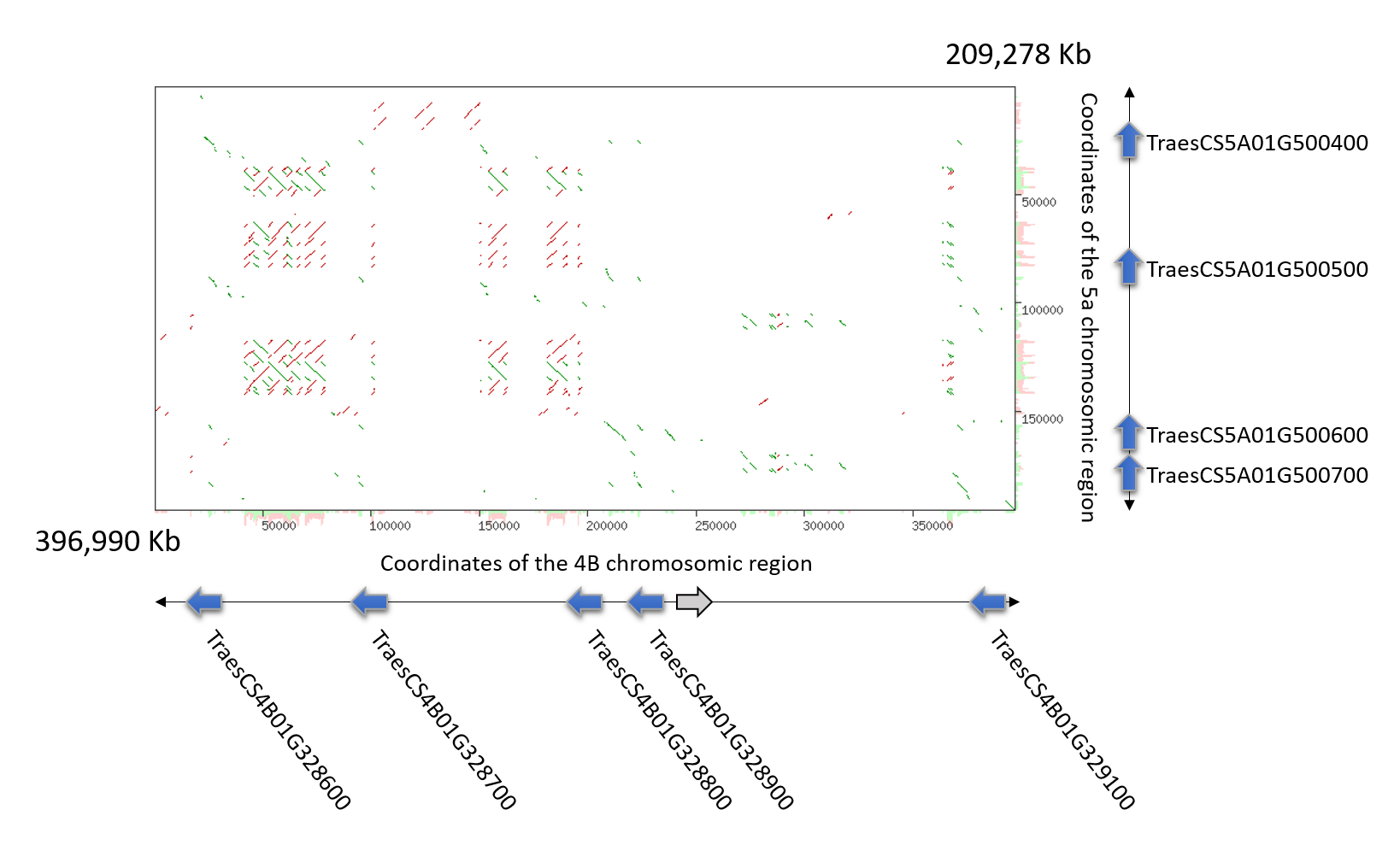

Supplement: S4 Fig — Each gene location is indicated on the x- and y-axis by a blue arrow. Green and red dots indicate sense and antisense homologies respectively. (TIF) [file pone.0213390.s004.tif]

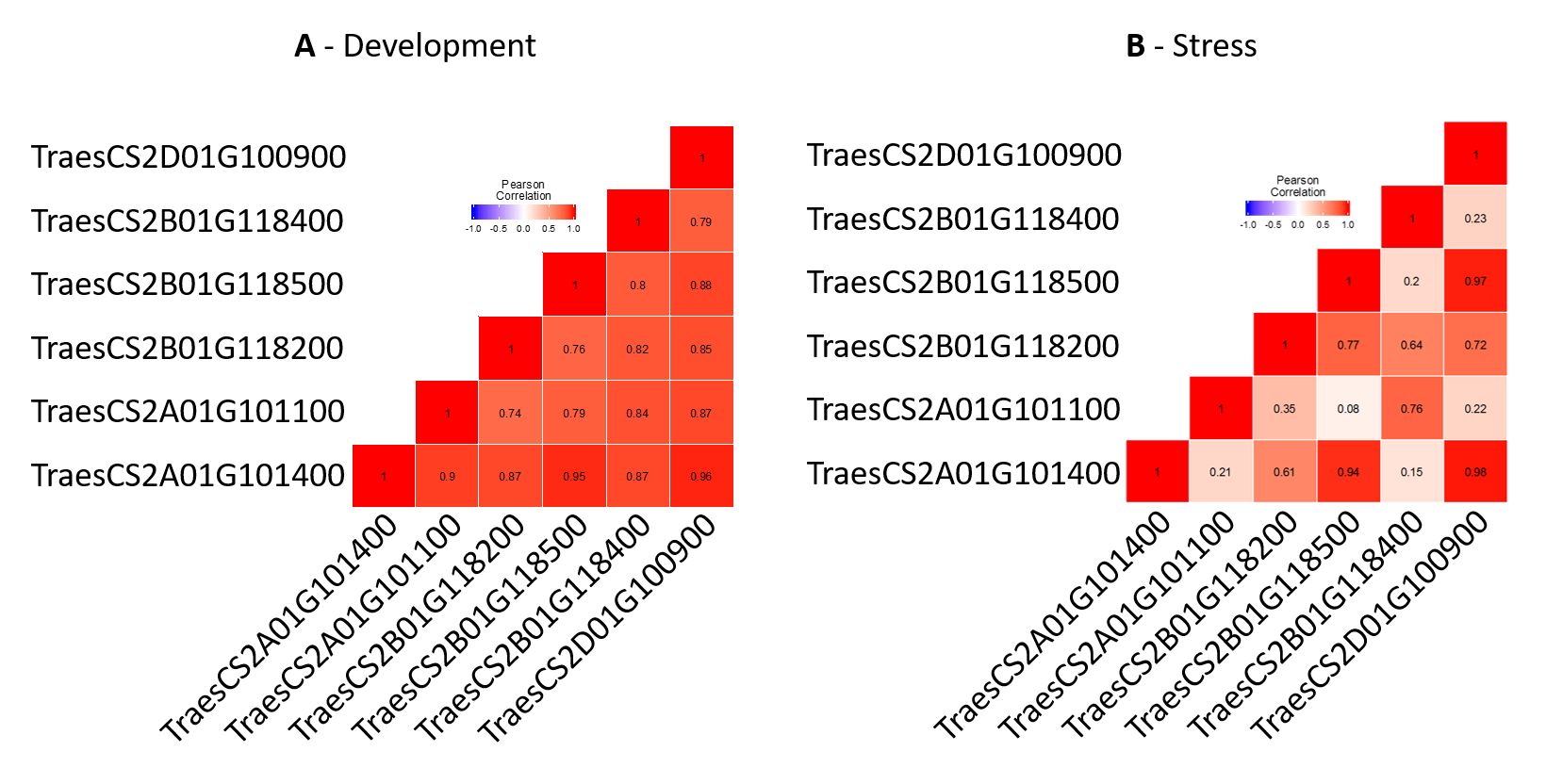

Supplement: S5 Fig — Pearson correlations between duplicated genes of the studied 2A-2B-2D chromosomal regions during development (A) and stress (B). (TIF) [file pone.0213390.s005.tif]

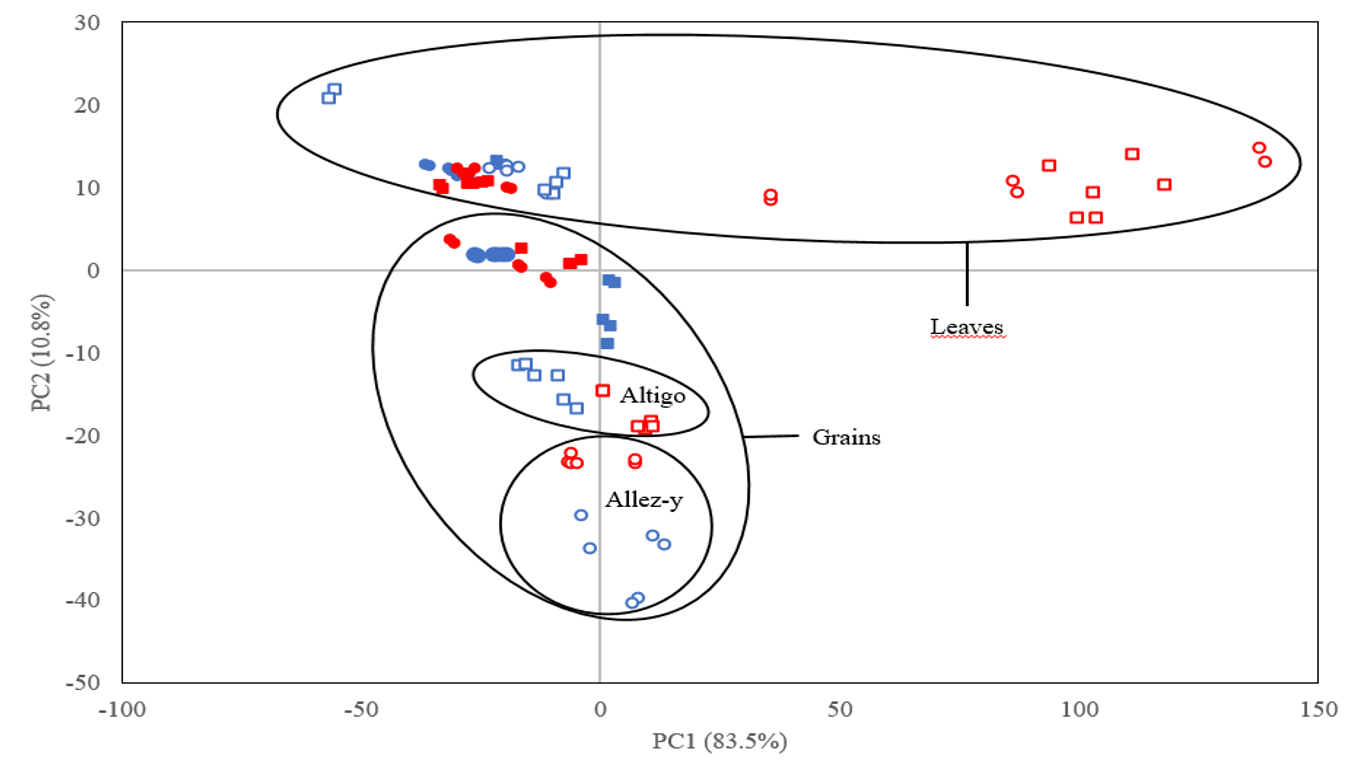

Supplement: S6 Fig — Expression of the 23 TaNAC genes in leaves and grains of Altigo (square) and Allez-y (circle) genotypes at 220°Cd (full form) and 450°Cd (empty form) in irrigated (blue) and drought (red) conditions. (TIF) [file pone.0213390.s006.tif]
